# SARS‐CoV‐2 infection associated acute kidney injury in patients with pre‐existing chronic renal disease: A report of two cases

**DOI:** 10.1002/iid3.333

**Published:** 2020-07-28

**Authors:** Yiru Wang, Yongman Lv, Qingquan Liu

**Affiliations:** ^1^ Department of Nephrology, Tongji Hospital, Tongji Medical College Huazhong University of Science and Technology Wuhan China; ^2^ Department of Health Management Centre, Tongji Hospital, Tongji Medical College Huazhong University of Science and Technology Wuhan China

**Keywords:** acute kidney injury, chronic renal disease, coronavirus, COVID‐19, SARS‐CoV‐2

## Abstract

**Background:**

The 2019 novel coronavirus (severe acute respiratory syndrome coronavirus 2 [SARS‐CoV‐2]) is driving a novel atypical pneumonia (coronavirus disease 2019 [COVID‐19]) outbreak in Wuhan, causing huge public health challenges both in China and globally. Limited data are available for information and prognosis on COVID‐19 patients with pre‐existing chronic kidney disease.

**Case presentation:**

Here we described the clinical characteristics and outcomes from two patients—a female aged 40‐year‐old and an 83‐year‐old male—who were subjected to SARS‐CoV‐2 infection, with history of chronic renal insufficiency. The female was admitted for dry cough and shortness of breath and the male was admitted for fever. The thorax computed tomography revealed patchy consolidation and ground‐glass opacity in both scattered lobes and the throat swab sample for coronavirus nucleic acid was positive. They were diagnosed with COVID‐19 and their renal function became progressively worse after infection with COVID‐19. After symptomatic support treatment, in both the patients, renal function was obviously restored, and both recovered from this pneumonia and conformed to the discharge criteria.

**Conclusion:**

SARS‐CoV‐2 infection may aggravate renal function impairment. It is crucial to monitor changes of renal function in patients with COVID‐19, especially those with primary kidney disease. Kidney protection interventions should be taken as early as possible, thereby improving the prognosis of patients with COVID‐19.

## 1 BACKGROUND

Since December 2019, there have been cases of atypical pneumonia caused by a novel coronavirus (severe acute respiratory syndrome coronavirus 2 [SARS‐CoV‐2]) reported in China and spreading to more than 20 countries around the world.^1^ With more than 65 000 cases and over 1200 deaths, predominantly in China but spreading, coronavirus disease 2019 (COVID‐19) disease poses huge challenges to global public health,^2^ especially as no specific treatment is available. The virus can cause severe respiratory illness, similar to SARS and Middle East respiratory syndrome (MERS).^3^

Coronavirus action into cells is through the ability of the virus to bind to the angiotensin‐converting enzyme 2 (ACE2) receptor in humans.^4^ ACE2 receptors are highly expressed in the lung, ileum, heart, and kidneys,^5^ and these organs should be considered as being targets for SARS‐CoV‐2 infection. Prior studies have noted that SARS and MERS can cause heart damage and liver damage.^6^, ^7^ A retrospective analysis showed that SARS can cause elevated serum creatinine and acute tubular necrosis, implying renal function damage in patients infected with SARS‐CoV.^8^, ^9^ A recent epidemiological study revealed that patients infected by SARS‐CoV‐2 presented with abnormal liver function, and acute kidney injury (AKI).^10^ AKI is associated with the development of high‐risk mortality, adverse outcomes, and longer intensive care unit stays.^11^ There are few detailed reports of renal function injury in relation to SARS‐CoV‐2 infection complicated with chronic nephropathy. Here in this study, we describe the clinical features of two patients with deterioration of renal function, who were diagnosed with COVID‐19.

## 2 CASE PRESENTATION

### 2.1 Clinical presentation and diagnostic findings

We did a retrospective review of medical records from two patients with COVID‐19 admitted to Tongji Hospital of Huazhong University of Science and Technology from 26 January to 31 January 2020. Diagnosis of COVID‐19 was based on the *New Coronavirus Pneumonia Prevention and Control Program* (5th edition) published by the National Health Commission of China.^12^ We also summarized the clinical data of two previous studies to extract the incidence of abnormal renal function or kidney damage in patients infected with SARS‐CoV‐2.^2^, ^10^, ^13^, ^14^

The sample comprised one man and one woman. They were admitted for fever or cough, and were diagnosed with COVID‐19 according to the clinical history, thorax computed tomography (CT) findings and positive of SARS‐CoV‐2 nucleic acid from throat swab sample. After admission, the baseline characteristics and laboratory findings were recorded, as summarized in Table 1. Patient 1 was treated with a broad‐spectrum antibiotic, ambroxol hydrochloride, recombinant human interferon α1b and methylprednisolone, and continuous renal replacement therapies (CRRTs), and the treatment regimen for patient 2 was similar to that of patient 1, except for renal replacement therapy. Both the patients made satisfactory recovery, with a significant improvement in serum creatinine levels. They recovered from COVID‐19 and complied with the discharge criteria of COVID‐19.

**Table 1:** Symptoms and laboratory test of two end‐stage renal disease patients with COVID‐19 at admission

Symptoms			Laboratory test		
	Patient 1	Patient 2		Patient 1	Patient 2
Fever >38°C	−	+	Total white cell count, ×10^9^/L	15.49	3.82
Chills or rigors	−	−	Lymphocyte count, ×10^9^/L	1.01	1.39
Cough	+	−	Neutrophil count, ×10^9^/L	13.25	2.04
Sputum	−	+	Hemoglobin, g/L	242	100
Haemoptysis	−	−	Potassium, mmol/L	4.83	6.29
Shortness of breath	+	+	Urea nitrogen, mmol/L	33.71	27.49
Malaise	+	+	Creatinine, mmol/L	1175	426
Anorexia	−	+	Albumin, g/L	29.2	36
Diarrhea	−	+	Alanine aminotransferase, U/L	11	17
Runny nose	−	−	Lactate dehydrogenase, U/L	474	223
Sore throat	−	−	C‐reactive protein, mg/L	180.5	42.1
Headache	−	−	Interleukin‐6, pg/mL	124.9	16.33
Dizziness	+	+	Interleukin‐2 receptor, U/mL	1622	1030
Weak	+	+	Tumor necrosis factor α, pg/mL	12.7	13
Edema	−	−	Urine protein	…	2+

Abbreviation: COVID‐19, coronavirus disease 2019.

### 2.2 Patient 1

**Figure 1:**
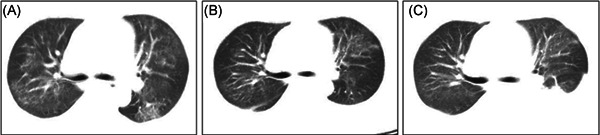
A, Serial chest computed tomography (CT) on initial presentation. B, CT scan of the thorax on day 7 showing patchy ground‐glass and nodal shadowing in both the left and right higher lobes slightly better than the first CT results. C, CT scan of the thorax on day 14 showing improvement of lung shadows after therapy, but there was pleural effusion

Patient 1 was a 40‐year‐old female, who was admitted to the Tongji Hospital on 26 January 2020 because of dry cough and shortness of breath for 1 week, and had a history of chronic glomerular nephritis. She also had dizziness and felt weak and malaise (Table 1). During the past 1 month, her serum creatinine level remained around 170 mmol/L, estimated glomerular filtration rate (eGFR) about 25 mL min^−1^ 1.73 m^−2^. Her urine volume was about 1500 mL per day. She had no other comorbid diseases except hypertension. The characteristics of thorax CT are shown in Figure 1A, which revealed patchy consolidation and ground‐glass opacity in both scattered lobes. After admission, routine blood biochemistry assays revealed total white cell counts and neutrophils counts were elevated, leukomonocyte counts were descended. Her serum creatinine levels were 1175 µmol/L and blood urea nitrogen was 33.71 mmol/L. The potassium and sodium levels were within a normal range. The patient was treated with a broad‐spectrum antibiotic, ambroxol hydrochloride, and methylprednisolone every 24 hours. Because renal function became progressively worse, on day 5, she was given CRRTs to protect kidney function. One week after treatment, her respiratory symptoms improved significantly and re‐examined chest CT showed shadowing in both lobes, which was slightly better than the first CT results (Figure 1B,C). Since two consecutive nucleic acid assays for SARS‐CoV‐2 were negative, she was conformed to discharge criteria and her serum creatinine levels decreased significantly.

### 2.3 Patient 2

Patient 2 was an 83‐year‐old male, who had chronic renal insufficiency for 5 years, and his serum creatinine was 254 μmol/L 1 month before admission. He was admitted to Tongji Hospital on 29 January 2020 because of having fever for 7 days. The patient also had diarrhea, anorexia, and malaise, but had no symptoms of cough or palpitation (Table 1). He had other comorbid diseases including hyperuricemia and gout for over 30 years. After admission, his laboratory data at presentation revealed lymphocytopenia with a normal total white cell count. His liver enzymes, total protein, and albumin were normal. Serum creatinine levels were 426 μmol/L, blood urea nitrogen was 27.49 mmol/L, and eGFR was 10.4 mL min^−1^ 1.73 m^−2^. The potassium level was 6.29 mmol/L, and sodium, calcium, phosphate, and magnesium levels were all normal. After treatment with a broad‐spectrum antibiotic and short‐term small dose of hormone, he did not develop respiratory failure or become critically ill. His serum creatinine gradually returned to their base levels (426 to 396 to 205 μmol/L) without using of a drug to reduce blood creatinine. The eGFR was 25.1 mL min^−1^ 1.73 m^−2^ on day 9.

Furthermore, to strongly determine whether SARS‐CoV‐2 infection affected renal function or kidney damage, we reviewed the latest two relatively larger studies focused on the renal function of patients with COVID‐19. The results of the two studies suggested that about 3% to 10% of patients infected with SARS‐CoV‐2 had abnormal renal function, including elevated creatinine or urea nitrogen. In addition, about 7% of patients experienced acute renal impairment (Table 2).

**Table 2:** Summary of renal function characteristics of COVID‐19 patients in two cohorts from published data

Characteristics	Cohort 1 (n = 99)^2^	Cohort 2 (n = 41)^13^
Age, y	55.5 (21‐82)	49 (41‐58)
Sex		
Male	67 (68%)	30 (73%)
Female	32 (32%)	11 (27%)
Laboratory findings		
Serum creatinine increased	3 (3%)	4/41 (10%)
Blood urea nitrogen increased	6 (6%)	N/A
Acute kidney injury	3 (3%)	3 (7%)

Abbreviation: COVID‐19, coronavirus disease 2019.

## 3 DISCUSSION

SARS‐CoV‐2 is a novel virus that can cause severe respiratory illness, acute respiratory failure, and other serious complications.^10^ The incidence of SARS‐CoV‐2 infections per 100 000 in men and women is 0.31 and 0.27, respectively, while the mortality rate is about 3.1%.^15^ SARS‐CoV‐2 is a coronavirus like SARS and MERS, and its genetic characteristics are 88% homologous with bat‐derived SARS‐like coronaviruses.^4^ It has been proved that SARS causes damage to organs other than the respiratory system. A study reported that among 536 patients with SARS, 6.7% developed AKI occurring at a median duration of 20 days (range 5‐48 days) after the onset of viral infection.^16^ Another report showed that 26.7% of patients with MERS developed AKI, and the median duration to the occurrence of AKI from symptom onset was 16 days.^9^ With the exception of a few clinical presentations, such as fever, cough, and dyspnea complicated by COVID‐19, impaired renal function with raised plasma creatinine is not a common finding in most patients with COVID‐19 at the time of the first clinical presentation. In the clinic, we observed that COVID‐19 could also lead to renal damage and proteinuria. A recent study focused on 59 patients with COVID‐19 and found that the majority of patients (63%) showed proteinuria, with modest levels of elevated plasma creatinine (19%) and urea nitrogen levels (27%), and CT scans revealed renal imaging abnormalities in all COVID‐19 patients.^17^

Here, we have reported two cases of acute‐on‐chronic renal failure in patients with novel COVID‐19 and chronic nephropathy. After being infected with SARS‐CoV‐2, their serum creatinine levels were significantly worse and had proteinuria. The serum creatinine level of patient 1 was 170 μmol/L 1 month before being infected with SARS‐CoV‐2, but this level increased to 1175 μmol/L after having the disease. Deterioration of renal function in both patients cannot be fully attributed to the progression of chronic renal disease itself alone, because they had six recent months where their renal function levels were relatively stable, although both had underlying renal disease. Moreover, the two patients' urine volume did not significantly decrease day‐on‐day, and within 1 to 2 months, such a significant change of renal function is rare in clinical practice. A study of patients with chronic kidney disease (stage IV) reported an average eGFR decline of 2.65 mL min^−1^ 1.73 m^−2^ per year.^18^ Patient 1 presented with no common clinical symptoms of uremia, such as fatigue, tiredness, pruritus, anorexia, or nausea.^19^ Although she had hypertension, her blood pressure remained within normal range. Patient 2 had significant hyperkalemia at the time of admission, but his serum kalemia level dropped to normal levels without reaching hyper levels again during hospitalization. Further, the serum creatinine levels of patient 2 declined steadily without using of lowering creatinine interventions. Overall, our data suggest that the possibility of acute deterioration of renal function caused by renal disease itself is very small, and that the SARS‐CoV‐2 virus infection promoted the deterioration of renal function in both the patients.

The exact mechanism of SARS‐CoV‐2 infection leading to acute exacerbation of chronic renal insufficiency is unclear. It has been shown that SARS‐CoV‐2 can specifically recognize and bind to ACE2 as a cell‐entry receptor, where the sequence of SARS‐CoV‐2 receptor‐binding domain is similar to that of SARS‐CoV.^4^, ^20^ ACE2 has been proven to be a functional receptor for SARS‐CoV, which contributes to the SARS coronavirus entering organs and causing multiple organ damage, such as acute pneumonia, acute diabetes, and gastrointestinal symptoms.^21^, ^22^, ^23^, ^24^ Previous studies have reported differential ACE2 expression in human tissues, where RNA‐seq showed that expression levels in the kidney were almost 100 folds than in the lungs.^5^ The results suggest that kidneys are susceptible to SARS‐CoV‐2. Development of acute renal failure during the SARS disease course is uncommon, but carries a high‐risk mortality (91.7%).^16^ In our study, both patients had been treated with broad‐spectrum antibiotic, and a short‐term small dose of methylprednisolone, and in addition, patient 1 received hemodialysis. Both patients recovered from COVID‐19, complicated by acute exacerbation of chronic renal insufficiency during the disease course, which carries a high‐mortality risk.

Another possible explanation for how SARS‐CoV‐2 infection leads to acute exacerbation of renal function is the role of cytokines. Cytokines such as interleukin (IL)‐6 and IL‐8 induced by SARS viral infections have been implicated as playing a critical role in AKI.^8^ These cytokines may increase during SARS‐CoV‐2 infection.^2^ As in our two cases, IL‐6, IL‐2 receptor, and tumor necrosis factor‐α expression levels were significantly increased, which were observed in a vast majority of other patients with COVID‐19. IL‐6 could bind to its receptor on mesangial cells and renal tubular epithelial cells, leading to the release of many other chemokines that recruit neutrophils and monocytes to the kidney,^25^ and then process and trigger AKI. Cytokine levels are useful in predicting mortality rates in patients with AKI who are critically ill.^26^ It is interesting to note that after treatment, both patients' kidney function partly recovered. This suggests that renal impairment due to SARS‐CoV‐2 infection is reversible. However, the rate of COVID‐19 with acute renal dysfunction is not clear. Therefore, further studies are needed to investigate the impact of SARS‐CoV‐2 on renal function, as well as the risk factors for renal impairment.

## 4 CONCLUSION

Taken together, these findings suggest that COVID‐19 may cause or accelerate renal damage. It is crucial to monitor changes of renal function in patients with COVID‐19, especially those with primary kidney disease or other high‐risk factors of kidney injury. Kidney protection interventions should be taken as early as possible, thereby improving the prognosis of patients with COVID‐19.

## AUTHOR CONTRIBUTIONS

YRW did the the data collection, literature review, and wrote the first draft of the manuscript. YRW and QQL analyzed the data and provided edits of the first draft of the manuscript. QQL and YML participated in the revision of the manuscript. QQL approved the final version.

## ETHICS APPROVAL AND CONSENT TO PARTICIPATE

The study protocol was approved by Medical Ethical Committee of Tongji Hospital. All participants gave written informed consent before inclusion and could withdraw at any time. Personal information was treated confidentially.

## CONSENT FOR PUBLICATION

The patients had been given written consent for their personal or clinical details along with some identifying images to be published in this study.

## Data Availability

The datasets generated and analyzed during the current study are available from the corresponding author on reasonable request.
